# A Rare Case of Warm Autoimmune Hemolytic Anemia With Intravascular Hemolysis: A Case Report

**DOI:** 10.7759/cureus.83916

**Published:** 2025-05-11

**Authors:** Saviz Saghari, Mason Arbabi, Olaniyi Fadeyi, Yunefi Wei

**Affiliations:** 1 Internal Medicine, West Anaheim Medical Center, Anaheim, USA; 2 Internal Medicine, University of Kentucky, Lexington, USA; 3 Hematology and Oncology, West Anaheim Medical Center, Anaheim, USA

**Keywords:** autoimmune disorder, autoimmune hemolytic anemia (aiha), igg autoantibodies, intravascular hemolysis, warm antibody autoimmune hemolytic anemia, warm autoimmune hemolytic anemia

## Abstract

This case report highlights a rare presentation of warm autoimmune hemolytic anemia (AIHA) with an atypical manifestation of intravascular hemolysis. The case underscores the diagnostic complexity and therapeutic challenges involved when AIHA presents with unusual features in the context of multiple underlying risk factors like infection, autoimmune markers, and occupational exposure.

A 53-year-old male with a history of mining-related environmental exposure and a single previous episode of syncope presented to the emergency department with weakness, fatigue, and a syncopal event. Laboratory findings revealed severe anemia with hemoglobin of 4.4 g/dL, elevated lactate dehydrogenase, low serum haptoglobin, and positive direct antiglobulin test (DAT) for IgG, consistent with warm AIHA. Intravascular hemolysis was noted despite a negative DAT for complement C3 and normal complement levels. The patient was treated with high-dose corticosteroids and blood transfusions, resulting in stabilized hemoglobin levels and discharge in stable condition. Further outpatient follow-up was recommended to evaluate potential underlying autoimmune or neoplastic etiologies.

This case highlights the importance of considering atypical mechanisms in warm AIHA presentations. Factors such as high antibody titers, IgG subclass variations, connective tissue disorders, infection, and potential neoplastic processes should be explored. This case contributes to the growing understanding of AIHA variants and underscores the need for individualized assessment in cases with complex presentations.

## Introduction

Warm autoimmune hemolytic anemia (AIHA) is typically characterized by extravascular hemolysis mediated by IgG antibodies, leading to red blood cell (RBC) destruction primarily in the spleen and liver. In rare cases, however, warm AIHA can present with predominant intravascular hemolysis, an atypical manifestation of the disease. Here, we describe a case of profound intravascular hemolysis in the context of warm AIHA, with an unusual clinical course that included severe anemia, syncope, and features suggestive of an autoimmune disorder. This case contributes to the literature by highlighting diagnostic and therapeutic challenges in diagnosing and managing intravascular hemolysis within warm AIHA.

## Case presentation

Patient information

The patient is a 53-year-old male with a history of occupational exposure as a former miner, with significant environmental exposure to coal dust. His only notable medical history included a single episode of syncope in January 2024, during which diagnostic workup revealed 59% stenosis of the left carotid artery with no other notable findings. At time of presentation, he was not on any medications for chronic conditions and reported no current alcohol or tobacco use, or use of any recreational substances. He did report a history of acute respiratory distress syndrome (ARDS) in the past related to his work as a miner, though details of this diagnosis were unclear.

Chief complaint

The patient presented to the emergency department (ED) after an episode of syncope at home, during which he experienced sudden weakness, fatigue, and jerking movements of the upper extremities. He reported urinary incontinence associated with the syncopal event, with orange-colored urine. There was no report of tongue biting, and he reported no prior history of seizures.

In the preceding days prior to his presentation, he had experienced progressive weakness, fatigue and exertional dyspnea, limiting him to walking only a few steps before feeling exhausted. He also reported a three-day history of subjective fevers and a cough.

Relevant history

Aside from his prior syncope in January, the patient's medical history was unremarkable, with no reported episodes of gastrointestinal bleeding or other signs of overt bleeding. He is an ex-smoker with 20 pack years.

Diagnostic assessment

Upon presentation, the patient’s vital signs were notable for tachycardia (heart rate 128 beats per minute), mild hypoxia (SpO₂ 94% on room air), and a blood pressure of 129/61 mmHg. Laboratory workup revealed severe anemia with hemoglobin at 4.4 g/dL, significantly elevated lactate (4.4 mmol/L), and leukocytosis (white blood cell (WBC) 16.3 x 10^3/μL). Liver function tests were also abnormal, with an elevated aspartate aminotransferase (AST) of 76 U/L and low albumin at 2.8 g/dL. The urinalysis (UA) was remarkable for 3+ blood and numerous red blood cells (RBCs), consistent with hematuria/hemoglobinuria. A peripheral blood smear showed severe anemia with nucleated erythrocytes and macrocytic changes, consistent with reactive inflammatory leukocytosis.

**Figure 1:**
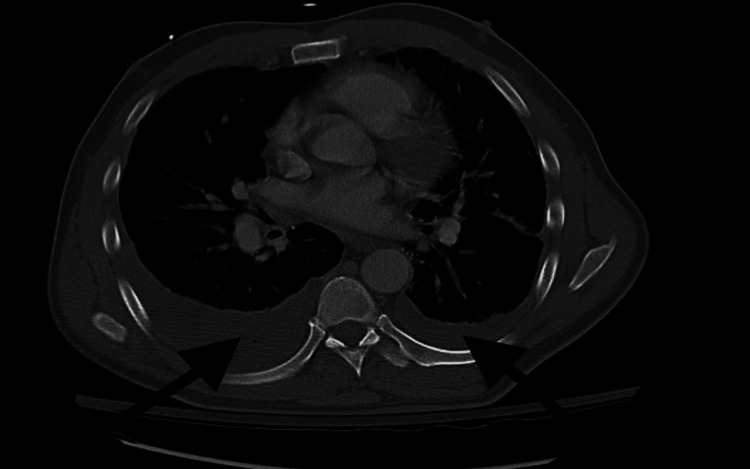
CT chest demonstrating bilateral pleural effusions (arrows), with the right side more prominent. These findings, along with mediastinal lymphadenopathy, raised concern for a potential underlying infectious, inflammatory, or neoplastic etiology.

A CT scan of the chest, abdomen, and pelvis revealed bilateral pleural effusions (Figure 1) with atelectasis, left lingula ground-glass opacities, and mediastinal and hilar lymphadenopathy, suggestive of a possible infectious or inflammatory process. CT head and neck demonstrated no acute findings. Although bilateral pleural effusions were observed, a diagnostic thoracentesis was not performed due to the patient's stable respiratory status and the non-loculated nature of the effusions.

Initial treatment involved orders for four units of packed RBCs (PRBC) and one unit of fresh frozen plasma (FFP); however, a positive antibody screen in the patient’s blood delayed the PRBC transfusion, requiring blood to be sourced from an external facility.

Hospital course

Following admission to the intensive care unit (ICU), the patient’s anemia was attributed to autoimmune hemolysis. His hemolysis profile was consistent with severe intravascular destruction, with lactate dehydrogenase (LDH) at 1500 U/L, low serum haptoglobin, and elevated indirect bilirubin. A direct antiglobulin test (DAT) revealed IgG positivity, indicative of warm AIHA. Notably, the DAT was negative for complement component C3. Cold agglutinin titer was negative <1:32.

The patient was treated with high-dose intravenous methylprednisolone for four days, followed by oral prednisone 40 mg daily as per hematology recommendations. Despite his unstable hemoglobin, which reached 3.9 g/dL, PRBC transfusion was initially delayed due to antibody-related issues. Eventually, he received four units of PRBCs and three units of FFP, which stabilized his hemoglobin levels to 9 g/dL (Table 1).

**Table 1:** Hemoglobin Trend During Hospitalization and Treatment Response PRBC: packed red blood cells

Day	Intervention	Hemoglobin (g/dL)
Admission		4.4
After steroids (Day 2)	IV methylprednisolone	3.9
After transfusions (Day 5)	4 units PRBC	9.0
Discharge	Oral prednisone	9.0

Further evaluation by gastroenterology included esophagogastroduodenoscopy (EGD) and colonoscopy, which showed nonspecific gastritis without other pathology. Infectious disease workup was significant for sepsis secondary to likely pneumonia, as evidenced by pulmonary findings and elevated inflammatory markers. This was treated with a five-day course of IV ceftriaxone and azithromycin.

Follow-up and outcomes

Throughout his inpatient stay, the patient’s hemoglobin level stabilized and he was eventually transitioned to oral corticosteroids for outpatient management. At discharge, he was hemodynamically stable with a hemoglobin level of 9 g/dL, and arrangements were made for outpatient follow-up and further hematologic evaluation. A lymph node biopsy was recommended given the patient’s history of mining-related environmental exposure and mediastinal lymphadenopathy, raising the possibility of underlying malignancy or an autoimmune process contributing to his presentation.

Prognostic characteristics and follow-up

Although the etiology of the patient's hemolytic anemia remained inconclusive, his autoimmune profile revealed positive antinuclear antibodies (ANA) with elevated ribonucleoprotein (RNP) and Smith antibodies. Additional autoimmune markers were negative. His complement levels (C3 and C4) were within the lower end of the normal ranges (C3 84 mg/dL and C4 15 mg/dL), making a typical complement-mediated process less likely.

## Discussion

Warm AIHA generally results in extravascular hemolysis by Fc receptor (FcR)-mediated immune clearance and is characterized by spherocytes on the blood film [1]. In a typical case of warm AIHA, IgG-coated red RBCs are recognized and phagocytosed by macrophages in the spleen and liver. However, this case of warm AIHA is notable for the predominance of intravascular hemolysis, as evidenced by severe anemia, hemoglobinuria, indirect hyperbilirubinemia and low serum haptoglobin, and without evidence of substantial complement involvement. Several mechanisms may account for the predominance of intravascular hemolysis in this case, which we discuss below.

The first is related to properties or concentrations of IgG antibodies. High concentrations, subclass variants or atypical properties of IgG autoantibodies could contribute to intravascular hemolysis.

High titers of IgG autoantibodies can be directly related to hemolysis. In previous studies of patients with IgG antibodies alone, the presence of hemolysis was predominantly dictated by the amount of RBCs bound to IgG [2,3]. When titers of IgG are 100, around 78% of patients experience hemolysis, and this increases to 94% when the titer is greater than 300. Additionally, high titers of IgG, which are found in intravenous immunoglobulin (IVIG) products can induce intravascular hemolysis [4].

Another factor that may contribute to intravascular hemolysis is the specific subclass of IgG involved, which can impact complement activation and cell destruction. Larger concentrations of RBC bound IgG are also associated with the presence of subclasses such as IgG1 and IgG3 which have been found to have a positive association with intravascular hemolysis [1]. IgG1 and IgG3 are effective in initiating complement pathways, potentially leading to partial or localized complement activation insufficient to deplete C3 and C4 levels but enough to cause intravascular hemolysis [5].

IgG antibodies of different specificities and subclasses have been shown to act synergistically in effecting antibody-dependent cellular cytotoxicity (ADCC), a mechanism where immune cells (like natural killer cells) are recruited to kill cells that are tagged with antibodies [6]. If multiple IgG antibodies with different specificities or subclasses are bound to RBCs, they could potentially act in concert to enhance the destruction of RBCs through this mechanism.

Another possible contributing factor to the case presentation is the underlying possibility of immune dysregulation secondary to an underlying connective tissue disorder. AIHA occurs in about 10% of systemic lupus erythematosus (SLE) patients and has been reported as the initial presentation of SLE in both adults and children [7,8]. In the present case, C3 and C4 levels were at the lower end of normal, which could be indicative of a partial or limited complement involvement, insufficient to deplete systemic levels.

In addition to autoimmune dysregulation, infections such as pneumonia could have exacerbated the hemolytic process in this patient. The patient’s history of fever, cough, and radiological findings consistent with pneumonia suggest that an underlying infection or inflammatory condition could have triggered the development of secondary warm AIHA. Pneumococcal infections in particular are a recognized cause of warm AIHA [9].

Pneumococci produce neuraminidase, an enzyme that cleaves N-acetyl neuraminic acid from cell surfaces, exposing the Thomsen-Friedenreich (TF) antigen, a component on the surface of erythrocytes. Exposure of the TF antigen can lead to various abnormalities, including hemolytic anemia and hemolytic uremic syndrome, following invasive pneumococcal infection [9].

Additionally, the patient’s smoking history and occupational exposure as a coal miner, along with mediastinal and hilar lymphadenopathy, raise concerns about a potential neoplastic process. Although studies on cancer outcomes in coal miners show mixed results, some research has indicated a link between coal mining and increased risks of lung and stomach cancer [10,11]. AIHA can also occur as a paraneoplastic phenomenon, especially in lymphoproliferative disorders, but associations have also been observed with solid tumors, including non-small cell lung cancers. Notably, among solid tumor cases associated with AIHA, approximately 70% presented with warm antibody AIHA, with lung cancer cases predominantly linked to the warm AIHA subtype [12].

One strength of our approach in this case was the comprehensive and multidisciplinary evaluation, which included input from hematology, infectious disease, and gastroenterology. This enabled a thorough assessment of potential underlying etiologies, including autoimmune, infectious, and neoplastic causes. Another strength was including an evaluation of the patient’s environmental and occupational history, allowing the consideration of broader factors that may have contributed to his presentation, such as potential neoplastic processes.

Compared to classic warm AIHA, which typically features extravascular hemolysis with mild anemia and splenomegaly, this case was unusual for its intravascular features, marked anemia, and lack of complement involvement. Such presentations warrant expanded diagnostic evaluation for autoimmune, infectious, or neoplastic contributors.

While the multidisciplinary approach provided valuable insights, there were also limitations in the diagnostic process that are important to consider. These include the unavailability of certain specialized laboratory tests, such as IgG subclass profiling, which could have provided more immediate insights into the mechanism of hemolysis. Additionally, while we recognized the importance of a lymph node biopsy given the patient’s lymphadenopathy and occupational exposure, this was deferred to outpatient follow-up, leaving uncertainty regarding a possible underlying malignancy. These limitations underscore the challenges of managing complex AIHA cases with atypical features in a real-world setting.

## Conclusions

This case underscores the importance of considering atypical intravascular presentations in warm AIHA. While autoimmune markers and radiological findings raised suspicion for a possible underlying systemic disease or malignancy, no definitive diagnosis was confirmed during admission. This case highlights the diagnostic complexity of AIHA and reinforces the need for IgG subclass testing, long-term follow-up, and individualized management strategies when faced with complex hemolytic patterns.
